# Transactions of the Amer. Med. Ass., for 1850

**Published:** 1851-03

**Authors:** 


### Part 2—Reviews and Notices of New Works.

ARTICLE I.

### TRANSACTIONS OF THE AMERICAN MEDICAL ASSOCIATION, FOR 1850.

The proceedings of the meeting at Cincinnati in May last, were reported in the Journal for July; and reviews of two of the reports, those on "Medical Science," and "Practical Medicine," were published in our last issue, and it now remains for us to notice the other portions of the volume, which contains 499 pages octavo.

*The Report on Medical Education*, by Dr. Roby, Chairman, when presented was very severely criticised, and the opinions advanced condemned by some members of the committee who had not been consulted by the chairman, although he had sent it up with a request that it be laid before them before it should be presented. It may therefore be regarded as embodying the sentiments of the chairman alone.

After a rehearsal of the main features of the discussion on Medical Education as heretofore carried on by the Association and the profession at large, the report briefly examines the subject, under the heads of *Preliminary Education*, *Public Instruction*, and *The tests of admission into the Profession.*

The generally admitted fact that students in this country enter upon their studies without sufficient preliminary training claims an enquiry into the causes, which is made, with the conclusion that they are dependent upon the Profession, the Schools, and the Students themselves.

It seems to us, that one great difficulty in the way of improving the character and qualifications of those entering the profession, which has generally been overlooked, is the lukewarm attachment of physicians to their calling; which leads them to speak slightingly and often disrespectfully of it. This will necessarily drive from its ranks, young men of high aims and qualifications.

If we would take pains to lay before the young men of liberal education, in our respective communities, the high claims which medicine, as a noble science and a useful art undoubtedly possesses, and the wide field it opens for investigation, with the opportunities and prospects it affords for high and honorable distinction, it would do more to call into our ranks such as are worthy, than all the tirades against ignorance in students, and denunciations against medical schools for admitting the unqualified, that have issued from all the reformers and all the conventions of our times. While we continue to dwell upon the dark side of the picture in our representations of the profession to the world, we will continue to drive from it those who alone can elevate it, and leave the field with all its richness to those of more humble aims, and more limited qualifications.

And in reference to the education of those who are designed by their parents and guardians for the study of Medicine, there is a most important error generally committed. However limited the means of the student, a thorough college course of academical instruction is commenced, in which one routine is too generally followed, whether to qualify for Medicine, Divinity, Law, Literature, or Commerce. And after two or three years of laborious study, devoted to the acquisition of the dead languages, (which are generally soon forgotten,) they are deemed highly qualified, and ready to take the first rank among students of Medicine. Now we submit that this time is mostly wasted, and that if spent in as systematic a course of training, in the study of the important facts and truths of natural science, with simply knowledge enough of the languages to enable him to understand the derivation and meaning of terms, it would give the student a preliminary education that would do more toward qualifying him for understanding the principles of our science, and store his mind with facts that would aid him in his investigations, and continually be called into useful requisition in his subsequent practice.

As to public or college instruction for students, which contains some of the objectionable features of the report, the document does not seem to favor the wholesale charges of "school" badly situated, scantily officered, and meagerly equippesd, as heretofore made, and thinks if there are such, they should be pointed out. Allusion to the lengthening of the term of the college session in the "Model School," is also made, and a query instituted whether the amouut of instruction is increased or not.

An error in reference to colleges of Pharmacy, that called forth some criticism in the association, which made the statement that there were none in active operation, is corrected in the printed report, and an explanatory note appended.

The report only extends over eight pages of the volume.

*Report of the Committee on Medical Literature by* Dr. Stille, Chairman.

This is a well written document characterized by fairness, sound discretion, and an independent expression of opinion. Though from the extent of ground travelled over in a review of the Medical Literature of this country during a year, it could not be expected that all that might be worthy of consideration could be examined thoroughly, so as to be adjudged fairly; this report comes as near filling the design of the appointment of the Committee as could be expected, and is altogether the best one upon the subject that has yet been presented to the Association.

Thirteen pages are devoted to a notice of the Medical Journals, their merits and defects, with numerous suggestions on criticism.

Of the original books issued from the American Medical press during the year, the first volume of Dr. Drake's Treaties is very properly regarded as the most extensive and important. A well merited compliment is paid to the author's research, learning, and ability, as displayed in this great work.

The other original works noticed are, "The Physician and Patient," by Worthington Hooker M. D., of Conn. "Baths and the Watery Regimen," by John Bell M. D. "The Universal Formulary," by R. E. Griffith, M. D. "American Medical Formulary," by J. J. Reese M. D. "Minor Surgery," by Dr. Hastings, and "The Diseases of Infants," by Dr. Churchill.

After general considerations of the importance and nature of literature, the subject of copy rights, individual and international, is pretty fully discussed.

The report closes by the following resolutions.

*Resolved*, That the Association regards the cultivation of Medical Literature as essential to professional improvement, and as adapted to form one of the broadest lines of distinction between physicians and all pretenders to the name.

*Resolved*, That, in the opinion of this Association, it is equally the duty and the interest of the profession to sustain its periodical literature both by literary contributions and subscription.

*Resolved*, That, since literary excellence is best developed by literary studies, the formation of Medical Reading Clubs, after the plan set forth in this report, is urged especially upon physicians in places where the periodical and other medical publications of the day are not readily accessible upon other terms.

*Resolved*, That the Standing Committee on Medical Literature be instructed to report to the Association at its next meeting what American medical work, published during the year of their service, in their judgment is the most valuable, and that, with the consent of the Association, such work shall be formally proclaimed by the President.

*Resolved*, That State and local societies are hereby recommended to offer pecuniary rewards or other distinction for the best memoirs founded upon original observation.

*Resolved*, That Medical Colleges are hereby recommended to distinguish the best inaugural thesis of every year by a public announcement of its subject, and the name of its author, and by such other names as they may deem appropriate.

*Resolved*, That the sum of ONE HUNDRED DOLLARS, raised by voluntary contribution be offered in the name of this Association for the best *experimental* essay on a subject connected either with physiology or medical chemistry, and that a committee of seven be appointed to carry out the objects of this resolution; said committee to receive the competing memoirs until the first day of March, 1851; the authors' name to be concealed from the committee, and the name of the successful competitor alone to be announced after the publication of the decision.

Appended is a table of original articles of interest published in American Journals during the year.

The report of the Special Committee appointed to consider the means of improving our Medical Literature suggested in the report on Medical Literature for 1849, of which Dr. W. E. Homer was Chairman, closes with the following,

"*Resolved*, That in the opinion of this Association the only legitimate means within our reach for the encouragement and maintenance of a National Medical Literature, are to increase the standard of preliminary and professional education required of those who would enter the medical profession; to promote the circulation among the members of the profession of the medical journals of the day; to encourage the establishment of district medical libraries, and to induce every practitioner to cultivate, with care, the fields of observation and research that are within his reach."

Next follows the Memorial of the Association to Congress on the subject of an international copy right law as reported by the special Committee appointed for the purpose, composed of Drs. George B. Wood and Isaac Hays of Philadelphia. It is well drawn up and presents to Congress one of the most important measures that have claimed the attention of the Association. As showing the position of American Authors at present, and the necessity of a change of this condition, we quote the following paragraph:

"As the law at present stands, British literary works may be published in this country without any compensation to their authors. It is obvious that American works, supposing them to be of the same grade of merit, in order to compete with those from abroad, must be published on the same terms. The American author, therefore, can expect no compensation for his labors, unless these are better in themselves, or better fitted to our peculiar wants than the British. Thus, not only is our native literature without encouragement; it is really discouraged; and in the present state of the law, American authorship can never flourish, as it ought to do. We afford to the world the example of a nation repressing the struggles of its own aspiring literature; of a nation doing what best it may to nip in the bud those energies, which, if allowed to expand into full bloom, would do more than can be done by any other means to set off, in the eyes of the world and of posterity, its substantial greatness."

The Reports of the Committee on Publication and of the Treasurer follow.

*Report of the Committee on Public Hygiene*, Dr. JOSEPH M. SMITH, Chairman.

This is mostly devoted to a consideration of the causes of Typhus Fever.

The conclusions of the Committee appear to be that this disease originates in the poisonous effects of the excretions of the body, mostly from the skin and lungs; when concentrated, from persons in health, but more particularly from those affected with the disease.

Appended to the main report are two lengthy and well drawn up papers; one by Dr Edward Jarvis on the Sanitary Condition of Massachusetts, and the other by Dr. J. C. Symonds on the Hygienic Characteristics of New Orleans.

The paper by Dr. Jarvis, especially, is full of interest, and but for the want of space should receive an extended notice. From it we learn more fully the great importance of laws for the registration of births, marriages and deaths, in acquiring correct information of the sanitary condition of different localities.

As illustrative of this we quote two passages which give some idea of the kind of information that might be gained were such laws in force generally in this country. By a correct knowledge of the mortality from the various diseases in each district of country, much might be done to ascertain the causes and to avoid or remove them.

From an examination of the deaths in the cities and country in New York and Massachusetts Dr. Jarvis concludes that

"The diseases connected with respiration are much more prevalent in the country than in the cities of both States; being in Massachusetts an excess of thirty-two per cent., and in New York twenty-eight per cent, of the pulmonary diseases that are not of the endemic or epidemic class. *The difference in favor of the cities in respect to Consumption is still greater*, The deaths from this disease were thirty-eight per cent. more in the rural districts of both States than in Boston and New York."

This result is contrary to the general opinion.

But when better facilities are offered for investigation the conclusions are more important and correct. This is illustrated by the following:

"The sanitary inquiries made in England and France have discovered very great inequalities of life and health among people of different classes, and in different conditions. Thus, according to several tables in the 'Report on the Sanitary Condition of the Laboring Classes of England and Wales,' prepared by Edwin Chadwick, Esq., of London, in the families of the

| | | | | | |
|---|---|---|---|---|---|
| Prosperous classes | 1088 | died at an | average age | of 42.6 | years. |
| Middling classes | 4791 | " | " | 29 | " |
| Poor classes | 19,849 | " | " | 20.4 | " |

In the families of the more comfortably situated, only 20 per cent. died under the age of five years; while among the poor, fifty per cent. of those who died had not passed that age.

Among the prosperous, 46 in every 100, and among the poor only 8 in 100, lived to their 61st year.

I have had an opportunity to make some limited inquiries in this State relative to the life and mortality of the various classes of people; and these have led me here to the same results as Mr. Chadwick reached in England: that, wherever there is a diversity of outward circumstances, there is a diversity of vital force, a difference of health and of longevity; that external poverty is but a sign of inward poverty, of a weak body and feeble mental and moral power; and that, generally, what the world calls poverty, the want of estate, or destitution, is not a thing of accident, or of external circumstance, but it grows out of the man, and is a necessary consequence of the quantity of vital force that belongs to him —to his body and to his mind."

*The Report on Adulterrted Drugs &c* by Dr. HUSTON which follows is a very valuable document; showing that although we have stringent laws now in force regulating the foreign importation of Drugs &c., there is yet something necessary to prevent the knavery of their adulteration and sophistication in this country.

As of especial interest to our readers in the West, we quote the conclusions of the Committee and heartily recommend the suggestions to their consideration.

"Extensive inquiries among physicians, manufacturing chemists, and druggists have led to the following conclusions:—

1st. That the wholesale druggists in the large cities, equally in the South and West as in the Eastern States, who are not specially engaged in sellng nostrums, either as proprietors or agents, conduct their business on fair and honorable principles. As a general rule, they buy their choice chemicals from those who manufacture them, and either import other articles, or get them directly from those who do, and are always disposed to supply good articles to customers who are willing to pay a remunerating price. At the same time, many of this class keep inferior articles, which they dispose of for a corresponding price to physicians and storekeepers who insist on buying at reduced rates.

2d. That the inferior and adulterated drugs are chiefly disposed of in the southern and western portions of the United States—to the physicians and people residing in the small towns and villages, and sparsely populated districts. That in the large cities, particularly in the Atlantic States, bad drugs are, as a very general rule, dispensed only by inferior apothecaries.

There is ground to hope that we shall hereafter be protected from the introduction of spurious drugs from abroad; and, if effectual means can be devised to prevent their sophistication and sale at home, a great boon will be conferred on the community. It is not probable that this can be fully accomplished; but the evil may certainly be very much limited. How shall this be done? Various plans have been suggested, of which the following may be considered as the most important:—

1st. To apply to the State Legislatures to pass laws authorizing the appointment of inspectors, and making it a penal offence to deal in adulterated drugs and medicines.

It is difficult to understand why fraud in the manufacture and sale of medicines, which have so important an influence on the health and lives of the people, should not be punished with the same severity as debasing and counterfeiting money, which merely affects their pecuniary interests. The past history of State legislation, in relation to the practice of medicine, affords little hope, however, that any salutary laws on this subject can be procured in many or all of the States of the Union; and without a general concurrance of action, no good will be accomplished. It is to the members of our own profession, therefore, in conjunction with the respectable druggists and apothecaries, that we must look for whatever reformation is to be accomplished.

2dly. It has been suggested that physicians should feel it to be their duty to inspect the medicines in the drug stores from which they are in the habit of obtaining supplies for themselves or their patients. This would exercise a wholesome influence, if submitted to by the apothecary, and frequently performed by the physician, neither of which, however, is very probable. A more effectual plan, because of its being more likely to be carried out, would be for the various State Medical Societies annually to appoint a board of examiners, who should procure samples of different articles from the drug stores within their limits, analyze and otherwise examine them, and publish the results. If this were impartially and skilfully done, it would excite the ambition of the meritorious and control the less scrupulous.

Properly to carry out this plan, as well as for their own security in making purchases, physicians should become better acquainted with the physical character of drugs, and be able, with the assistance of a good treatise on Chemistry, to analyze the various chemical articles recognized in the Pharmacopœia. The requisite apparatus for this purpose, which need not be costly, should be in every physician's office, and good specimens of the various articles of the materia medica, with samples of the inferior or adulterated. This is especially desirable in offices into which students of medicine are received.

3dly. The co-operation of the druggists and apothicaries in discountenancing and putting down the traffic in inferior and adulterated medicines should be solicited. For this purpose, they should be encouraged to institute pharmaceutical associations in every considerable town throughout the country, which, more than anything else, would tend to elevate the professional and moral standing of their craft. Men who are in the habit of meeting together for laudable purposes are far less liable to plunge into bad practices than the isolated being whose better feelings are not warmed by association. The establishment of such societies has always been salutary. In Philadelphia, the institution of the College of Pharmacy, with its cabinets, its lectures, and excellent quarterly Journal, which is published regularly, has raised the character of the apothecaries to an enviable hight; and in the city of New York, where a like organization has been more recently formed, similar effects are observable.

4thly. In making their purchases of medicines, physicians should be willing to pay fair prices, and be careful to procure them only from the most respectable druggists. Men of this character, selling in large quantities, never demand exhorbitant profits, and it is not to be expected that they will sell good articles at a loss."

The report on *Indigenous Medical Botany*, Dr. Ives Chairman, is made up of two papers. One by Dr. Ives on the virtues of Isnardia palustris, Senecio aurens, Neottia pubescens, Cypripedium, Cornus circinata, Cornus florida, and the Epigra repens.

The other by Dr. Frost is made up of "Extracts from Dr. Barratt's notes on the Indigenous Plants of Abbeville district, S. C. The report altogether is brief but contains some useful facts and suggestions.

*The Report on Surgery* by Dr. Mussey, Chairman, is a very full account of the important suggestions, facts and operations that have transpired in this country during the year.

It is a model of what we conceive such a document should be, giving all that could be collected, that was deemed worthy by the author.

It extends through sixty pages of the report and is too multifarious in its topics to admit of analysis in our pages. Appended to it are three papers on Anæsthesia, one by Dr. J. C. Warren, one by Dr. S. D. Gross, and one by Dr. W. L. Atlee, and a paper on operations for the cure of Cancer by Dr. J. C. Warren.

In an appendix to the volume are, a paper read before the Association by Dr. N. S. Davis which was given to our readers in the last number of the Journal. A paper read before the Association by Dr. J. Evans on "The Obstetrical Extractor." "A Brief Notice of some of the Physicians of the U. S. who have died within a few years; by Dr. Stephen W. Williams," and a "Catalogue of the Officers and Permanent Members of the American Medical Association." E.

